# Exploring perspectives and insights of experienced voyagers on human health and Polynesian oceanic voyaging: A qualitative study

**DOI:** 10.1371/journal.pone.0296820

**Published:** 2024-04-15

**Authors:** Marjorie K. Leimomi Mala Mau, Sarah A. Stotz, Christina Mie Minami, Haunani Hiʻilani Kane, Kamanaʻopono M. Crabbe, Heidi Kai Guth

**Affiliations:** 1 Department of Native Hawaiian Health, John A. Burns School of Medicine, University of Hawaiʻi at Mānoa, Honolulu, Hawaiʻi, United States of America; 2 Department of Food Science and Human Nutrition, Colorado State University, Fort Collins, Colorado, United States of America; 3 Touro University Nevada College of Osteopathic Medicine, Henderson, NV, United States of America; 4 School of Ocean and Earth Science and Technology, Earth Sciences, MEGA Laboratory, University of Hawaiʻi at Mānoa, Honolulu, Hawaiʻi, United States of America; 5 Asian and Pacific Islander American Health Forum, Washington, DC, United States of America; 6 Native Hawaiian and Pacific Islander Affairs, Hawaiʻi Executive Collaborative, Honolulu, Hawaiʻi, United States of America; 7 Kai Hoʻoulu, LLC, Honolulu, Hawaiʻi, United States of America; German Archaeological Institute: Deutsches Archaologisches Institut, GERMANY

## Abstract

**Background:**

The Worldwide Voyage (WWV) was a 3-year (2014–2017) open-ocean voyage to circumnavigate the world using Indigenous knowledge and navigational skills aboard Hōkūleʻa, a traditionally designed Native Hawaiian (NH) voyaging canoe (waʻa kaulua). Each WWV segment included experienced crew and leadership who were recognized by their voyaging peers as highly experienced in Polynesian oceanic voyaging. This study explored the perceptions and insights of WWV-experienced ocean voyagers on the interconnection between human health and oceanic voyaging.

**Methodology:**

A constructivist approach with a storytelling-based moderator guide was used to conduct focus groups and informant interviews of experienced crew and voyaging leadership. Participants were interviewed and recorded transcripts were analyzed using content analysis. Triangulation of analysis included secondary thematic review by two independent NH cultural practitioners and participant member checking. Purposive sampling was used to enroll 34 of 66 eligible highly experienced voyagers (leadership n = 6; crew n = 28) in 5 focus groups and 4 informant interviews.

**Results:**

Six themes emerged: 1) Indigenous context (spiritual and natural environment); 2) Importance of relationships and community; 3) Description of life on the canoe; 4) Holistic health; 5) Mindfulness, stress reduction and emotional health; and 6) Opportunities for intervention. Themes 1–5 were inductive and intricately interrelated, and theme 6 was deductive in that it directly resulted from a moderator guide question. Theme 6 offers strategies to improve the impact of voyaging and health well beyond the physical voyage with recommendations for improved transition back to land and developing a waʻa community context, which reflects a traditional voyaging experience.

**Conclusions:**

Polynesian oceanic voyaging is strongly perceived as a positive and transformative holistic-health-promoting experience.

**Significance:**

Recommendations to promote generalizable health benefits of a voyaging lifestyle offers a promising and culturally grounded approach warranting future studies to understand mechanism and potential impact for improving health inequities.

## Introduction

Native Hawaiians (NHs), as Polynesians, first explored and settled the northernmost point of the vast Polynesian Triangle in the Pacific Ocean sometime between 1,100 and 890 years ago [1–8]. Over time, as voyages to and from Hawaiʻi and the South Pacific waned, NHs evolved as a distinct ocean-based people in the isolated ecosystem of the Hawaiian archipelago [1,2]. The Polynesian Triangle is geographically defined by Rapa Nui (a.k.a. Easter Island) in the southeast, Aotearoa (a.k.a. New Zealand) in the southwest and the Hawaiian archipelago in the north, and it encompasses more than 10 million square miles of the earth’s surface [9,10]. Today, many of the descendants of the first inhabitants of Hawaiʻi, NHs, are actively engaged in a robust and resilient revitalization of a growing number of cultural practices such as wayfinding (instrument-free navigation), kālai waʻa (traditional canoe building), nā holowaʻa (traditional ocean voyaging), hula (NH traditional dance form), and ʻolelo Hawaiʻi (NH language), among others [2,3,10–15]. Within Oceania, NHs and Pacific Islanders (PIs) share a common ancestry as people who explored and settled the Pacific with double-hulled voyaging canoes (waʻa kaulua), lived reciprocally with their island environments [1–5,10], and continue to experience generational harm from entrenched colonization from European and American intruders, dating back to the 1700s [13,15,16].

Consequential to foreign occupation in the Pacific and specifically in Hawaiʻi, the only Pacific Island that is an American state, came unfamiliar diseases (e.g., infectious disease) social discord (e.g., near-extinction of cultural practices, language) and political conflict (e.g., stolen sovereignty, land), until eventually the NH population census dropped precipitously to <10% of the pre-contact population [13,15,17,18]. Today, NHs and PIs continue to experience a disproportionate burden of health disparities. For example, NHs and PIs consistently report higher morbidity and mortality rates for diabetes mellitus, cardiovascular disease, and multiple forms of cancer (i.e., breast, colon, and lung cancer) [19–21]. These disparities occur at younger ages, approximately 10–15 years earlier than those of European derivation, and are often found with more advanced or severe forms of disease and associated comorbidities at diagnosis [19–25]. Multiple studies have reported the association of changes in dietary intake from a traditional diet to a western diet, and an active island-based lifestyle to a more sedentary one as primary risk factors [21,26–28]. Yet, attempts to identify genetic markers to explain the excess burden of disease has been largely unhelpful in explaining the persistent health disparities and has led to a greater appreciation and emphasis on understanding the complex nature of socioeconomic, environmental and other social determinants of health that likely contribute to socio-biological mechanisms underlying health inequities across diverse populations.

Recent studies suggest that socio-ecological factors (e.g., poverty, social networking) may potentially be key to understanding the pathophysiology of metabolic health disparities via epigenomics and gut microbiome shifts [29,30]. A growing number of NH researchers have maintained strong relationships with NH communities, built upon collaboration, trust and respect [31]. Drawing on the health benefits of a strong cultural identity and a strengths-based approach, NH scientists have forged ahead, supported by NH communities, to improve health and health care inequities using cultural practices, instead of only relying on interventions designed and tested on non-Indigenous peoples that generally have not been as successful for Indigenous populations [31,32]. One promising example includes NH cultural practices, such as hula, that have been shown to significantly reduce diastolic blood pressure compared with usual care [33,34].

**Fig 1:**
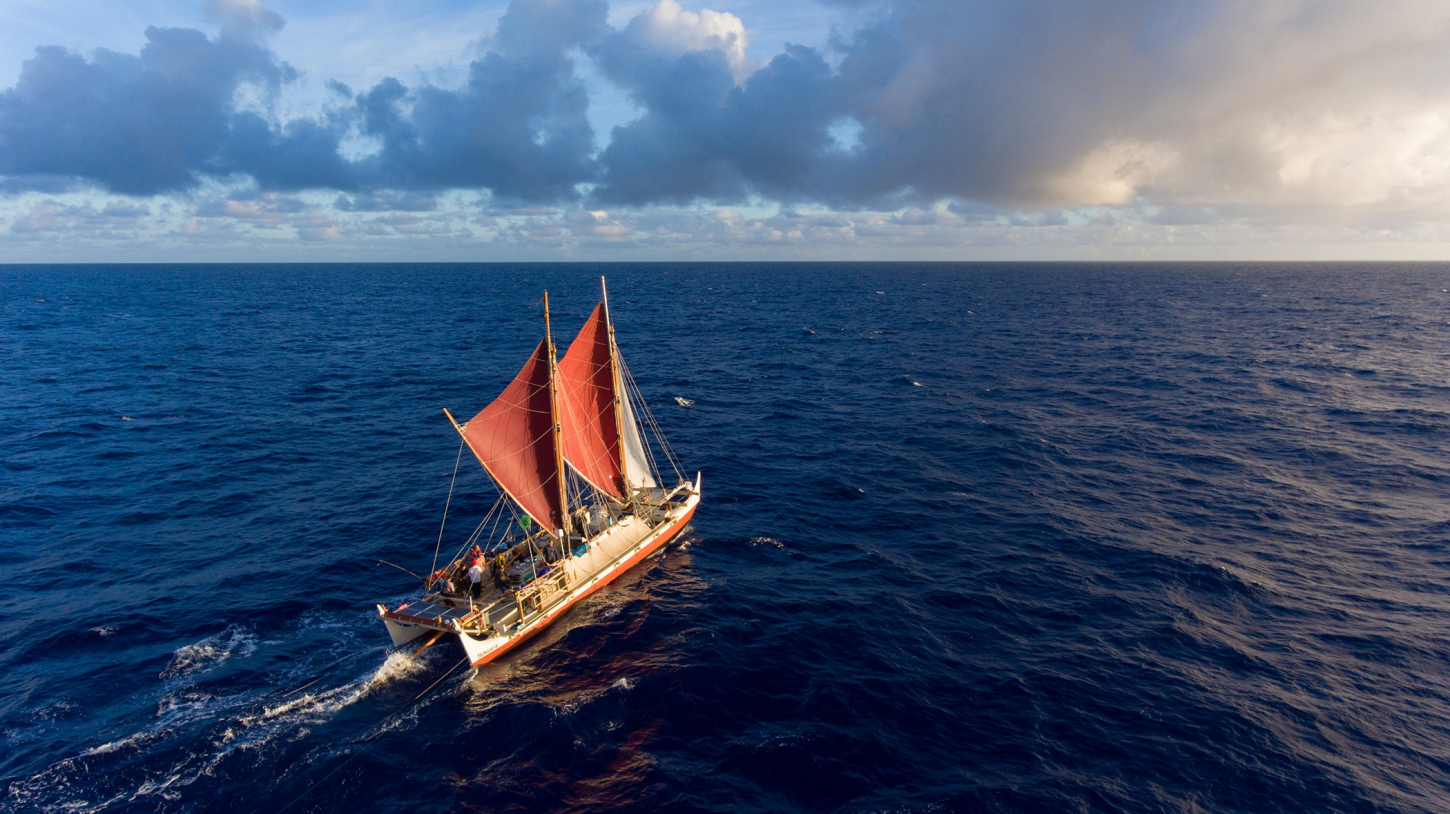
Photograph of Hōkūleʻa under traditional crab-claw sails during a deep-sea portion of the Polynesian Voyaging Society’s 2014–2017 Worldwide Voyage (WWV). Hōkūleʻa is 62 feet (18.9 m) long and 23 feet (7.01 m) wide, with 300 square feet (27.87 square meters) of deck space for an average crew of 12 people. All food and water is stored in the hulls, where crew also sleep—head to toe—under the tan canvas covers. The total covered sleeping area is approximately 138 square feet (12.82 square meters). Solar panels power safety lights and communications. Location information is held in the heads of traditional, non-instrument navigators. Crew fish during daylight hours, cook on a 2-burner propane stove, and wash (people, dishes and clothes) with ocean water. Hōkūleʻa has no engines, refrigeration, nails or screws. Approximately 5 miles (8.05 km) of rope lash her together, and at least 2 miles (3.22 km) of line make up her rigging. A traditional voyaging canoe, she is powered by the wind and steered with large paddles. Copyrighted photo courtesy of Naʻalehu Anthony.

By the 1970s, NHs were the most likely population in their homeland to be incarcerated, unemployed and at the bottom rungs of health and education; they were considered the least likely population to succeed in Hawaiʻi, and much of their culture verged on extinction [13–16]. The Native Hawaiian Renaissance that began in the early 1970s –in part with the birth of Hōkūleʻa, a replica of a traditional Polynesian voyaging canoe (**Fig 1**), resulted in a resurgence of cultural practices such as inter-archipelagic voyaging, traditional healing methods of hoʻoponopono (to reestablish balanced relationships) and lāʻau lapaʻau (use of medicinal native plants) as means to restore traditional NH cultural practices and ways of life [11–14,35]. NH leaders and activists acknowledge that connecting to one’s cultural identity requires access to healthy natural resources [14,15,36]. Similar to many Indigenous peoples, community health necessitates environmental health, in part because natural resources are needed for cultural practices, and because the genealogies of the people are tied to their human and environmental ancestors in storied and active ways [37–40]. To further understand and gain a deeper insight as to the health, cultural connection, potential benefits or risks associated with ocean voyaging, we enrolled experienced voyagers as recognized “experts” in the cultural practice of oceanic voyaging following their participation in Hōkūleʻa’s Worldwide Voyage (WWV) of 2014–2017 (the first global circumnavigation using traditional voyaging methods and a waʻa kaulua (NH double-hulled canoe)) [41]. **The purpose of this study is to explore the insights and perceptions of experienced ocean voyagers of the WWV on the interconnection between human health and ocean voyaging.**

## Methods

A constructivist epistemological approach was used to frame this multiple methods study [42]. This approach privileges the perspectives of unique individual voices by recognizing there is no universal truth (or correct answer) to multi-faceted, complex phenomena [43]. Thus, a diverse sample of interviewees, including both experienced crew and voyaging leadership, were enrolled to provide diversity in age, gender, and years of voyaging experience, allowing the researchers to triangulate perspectives to crystalize the validity and rigor of the findings [44].

### Participant recruitment and sampling

Researchers employed a purposive sampling approach [45] to recruit participants from a pool of experienced crew who voyaged during the WWV (2014–2017). Participants eligible for this study met all of the following criteria: 1) Qualified as deep-sea or open-ocean crew or at-sea leadership (e.g. watch captain, captain, navigator) as determined by voyaging peers; 2) Completed basic seamanship training; 3) Prior experience, as crew, aboard a double-hulled voyaging canoe in diverse ocean voyaging conditions; 4) Successfully passed physical agility, strength and endurance testing requirements; and 5) Significant experience conducting canoe maintenance work, which exemplifies the capacity for teamwork, leadership initiative, maturity and the development of skills to care for and maintain the canoe both on land and at sea. Recruitment was conducted by invitation to this limited pool. Eligible crewmembers were invited from Oʻahu and Hawaiʻi Island, as the majority of experienced crew from the WWV resided on those two islands. Of the 66 individuals who live on Oʻahu or Hawaiʻi Island and met the eligibility criteria, 34 (52%) participated. This study was submitted to the University of Hawaiʻi Committee on Human Subjects (protocol 2018–00934) and was approved as exempt from federal regulations documented in the Code of Federal Regulations 45 CFR 46.101(b)2. We obtained written informed consent from all participants at enrollment. All focus groups and informant interviews were conducted in-person. The recruitment and enrollment period was October 10, 2018 to July 24, 2021, inclusively.

Researchers held a total of four individual informant interviews and six focus groups. Each focus group consisted of 2–7 members and were conducted in person. Four individual interviews with voyaging leaders and one focus group that included only two voyaging leaders were conducted separately from crew to mitigate power dynamics between crew and leadership and to facilitate honest, safe focus group discussions. All interviews and focus groups were conducted by 2 trained moderators (HKG and/or MKM), using the same moderators guide developed by 3 of the co-authors based on cultural background, voyaging experience and prior research relevant to voyaging and health. (Moderators Guide—S1 File).

### Data collection

All participants completed a brief demographic and physical activity questionnaire. All interviews and focus groups were conducted using a narrative “talk story” format consistent with local NH and PI norms to create a safe cultural context to express personal opinions and insights and to foster trust [46,47]. Interviews used the same semi-structured moderator guide, consisting of five open-ended questions with additional probes aimed at soliciting further insights to personal or observed experiences on ocean voyaging, health and/or well-being [48]. Each interview or focus group lasted between 90 and 150 minutes and was audio recorded and then transcribed verbatim by a professional transcription company. The transcriptions were checked by a single researcher (HKG) for accuracy (grammar, spelling), cultural context, Hawaiian language and nautical terms, and cross-checked with field notes taken by at least one of the interviewers. The two voyaging leaders from the small focus group also reviewed, edited and verified the contemporaneous notes taken by one researcher, because the audio recording of their interview was partly inaudible.

### Analysis

#### Survey data

Demographic data was summarized using descriptive statistics. Self-reported physical activity levels were calculated according to the protocol for the Lipid Research Clinics (LRC) Physical Activity questionnaire [49]. Scoring of the LRC items identified individuals into 1 of 4 physical activity categories from “Very Low Active” (lowest physical activity) to “Very Active” (highest physical activity level) according to self-report. We dichotomized the participants according to age >50 years old (y.o.) and ≤ 50 y.o., as age is known to be correlated with level of self-reported physical activity (S2 File–Sociodemographic Questionnaire).

#### Description of voyaging days at sea by physical and mental endurance

**Fig 2:**
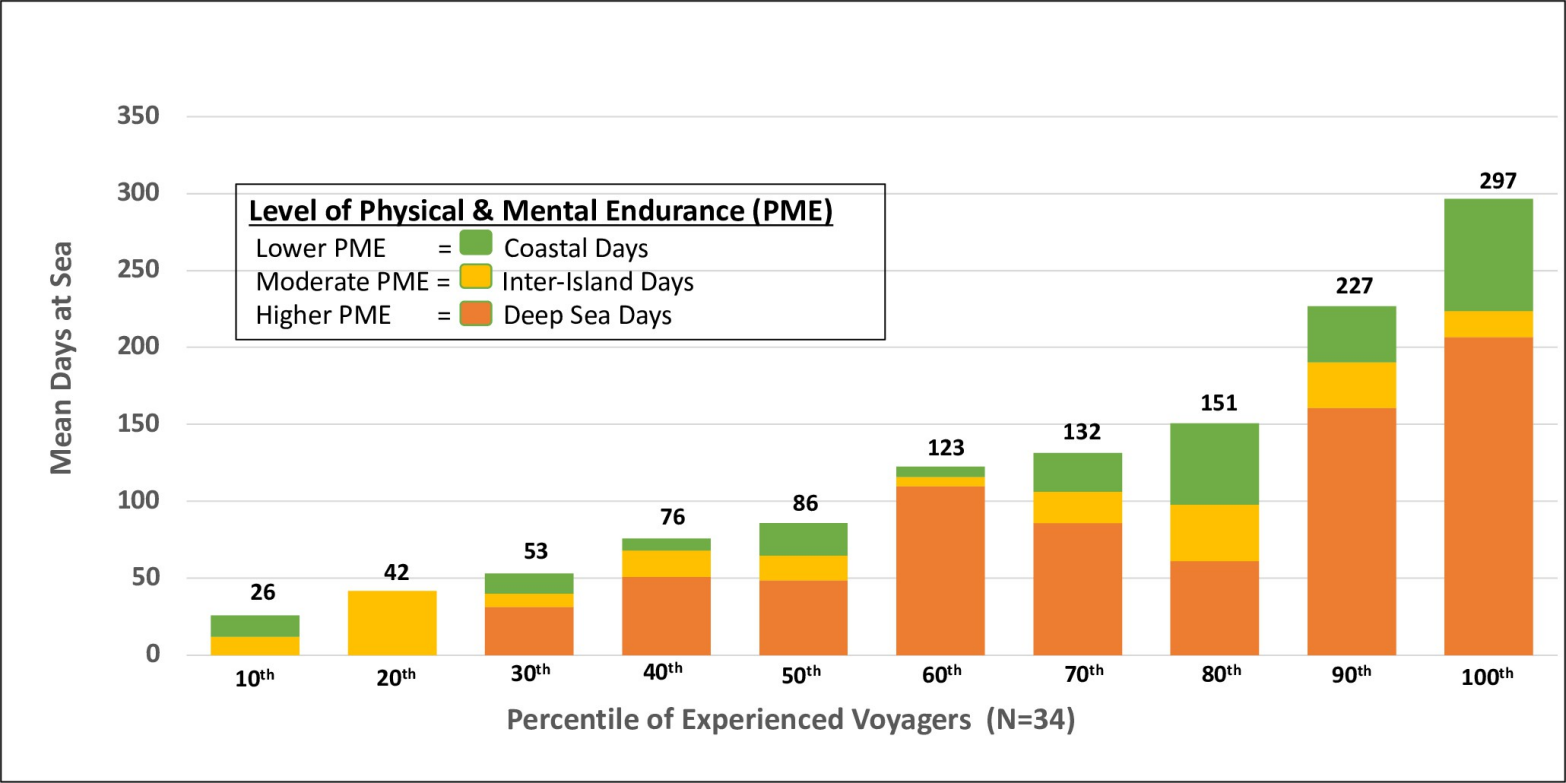
Summary of ocean voyaging experiences by experienced voyagers (n = 34, WWV 2014–2017).

Each leg or section of the WWV provided a unique set of voyaging circumstances such as weather conditions, crew members, leadership (e.g., captains), land destinations, and cultural context. Each leg was characterized into 1 of 3 types of voyaging according to physical and mental endurance (PME) levels, with deep sea days of highest PME, inter-island days of moderate PME and coastal days of lower PME level. The PME experienced by each voyager varies according to type of voyage leg. To compute the mean days at sea by type of voyaging days, all participants were ranked according to the total number of days voyaged into 10 sub-groups (deciles) from lowest (10%ile) to highest (100%ile) number of mean days at sea. Each decile represents the weighted-mean days at sea of approximately 3–4 individuals. PME-weighting was calculated for each decile by multiplying the mean days at sea by the PME factor for that type of voyaging leg per individual included in that decile. The PME factors for each type of voyaging segment were weighted as follows: Deep Sea = 1.0 PME factor; Inter-island = 0.8 PME factor; and Coastal = 0.6 PME factor. Thus, the PME-weighted “Days at Sea” represents the mean of all individuals for that decile stratified by type of voyaging and weighted by PME factor (**Fig 2**).

#### Qualitative (interview and focus group) data

The team employed an iterative qualitative content analysis approach for the qualitative data [50,51]. Two researchers (SAS, HKG) reviewed the transcripts to establish a codebook that included both deductive (*a priori)* codes (i.e., codes based on the moderator guide) and inductive codes (i.e., codes that emerged directly from the data). These researchers double coded 100% of the transcripts and met weekly to discuss such topics as coding discrepancies, harmonized use of the codes, and emergent themes [52]. Researchers chose to double code 100% of the transcripts because of the unique positionality of the researchers: one is an experienced deep-sea voyager, and the other a qualitative methodologist who had no previous knowledge of or training in voyaging. The content analysis approach included coding data, categorizing the codes, and reorganizing the categories into thematic representation through a series of assertions and interpretations [53]. Using this method, the primary coders compared data across transcriptions to find similarities and differences. The researchers used Atlas.ti (Version 8.1.1) to organize, sort, code, and store data, which helped to facilitate a transparent analytical process [54].

To further triangulate the analysis and crystalize integrity of findings, two NH cultural practitioners of different generations and genders with prior ocean voyaging experience provided secondary thematic review [44]. Researchers also employed member checking, using the draft themes with the interview and focus group participants. These draft themes were presented to the participants via individual email correspondence approximately 1–3 years following original data collection. A total of 56% (18 of 34 participants) provided active feedback to the member checking request. The participants’ member-checking feedback was incorporated into the final analysis and thematic findings.

## Results

### Survey findings

Of the 34 experienced voyagers, nearly two-thirds were NH or PI (65%), married (65%) and men (65%), with a mean age of 52.5 years (range 29–82 years) (**Table 1**). Educational attainment included 35% (n = 12) who had completed “graduate” degrees (masters or doctoral), 30% (n = 10) who had completed 4-year bachelor or 2-year associate degrees, and 35% (n = 12) who had completed high school up to “some college”. Level of physical activity by age revealed that the older voyagers (>50 y.o.) were uniform in reporting “moderate to very active physical activity” levels, while the younger voyagers (≤ 50 y.o.) reported a mix of 18% self-described as “very low physical activity” and the remaining 82% self-reporting as “moderate to very active” compared with their peers (**Table 1**). During the WWV, the range of total days at sea per experienced voyager varied from 29 to 476 days of voyaging, with an overall median of 104 days and a mean of 144 days at sea **(Table 1**). However, when viewed as PME-weighted, the mean “days at sea” by decile ranged from a mean of 26 days (10th%ile) to 297 days (100%ile) at sea. In terms of type of voyaging, 80% of experienced voyagers sailed deep sea days requiring higher PME, as compared with 20% of the cohort who sailed primarily on coastal and inter-island legs requiring lower to moderate PME (**Fig 2**). In summary, this cohort of experienced voyagers were among some of the most sea-worthy, dedicated and highly skilled oceanic voyagers among the >250 crew members who participated in the WWV. Thus, the insights and reflections of this group stems from their extensive involvement during the WWV as well as the many years of oceanic voyaging experience that preceded and followed the 2014–2017 WWV.

**Table 1:** Demographic characteristics of experienced ocean voyagers (n = 34).

Characteristic	N (%) or mean (range)
**Age** years (mean, range)	52.5 years (29–82)
**Women**	12 (35%)
**Race/Ethnicity** (n = 33)	
Native Hawaiian, Pacific Islander*	21 (64%)
European derived**	6 (18%)
Japanese	4 (12%)
Filipino	2 (6%)
**Married**	22 (65%)
**Education Attainment**	
High School—Some College	12 (35%)
College degree (Associates, Bachelors)	10 (30%)
Graduate degree (Masters, Doctoral)	12 (35%)
**Total Days at Sea per person** (mean/median/min-max)	144 / 104 / 29–476 days
**Usual Physical Activity Level** (self-reported)	
**Less than 50 years old (n = 17)**	
Very Low Active	3 (18%)
Moderate Active	2 (12%)
Very Active	12 (70%)
**More than 50 years old (n = 17)**	
Very Low Active	0 (0%)
Moderate Active	4 (23%)
Very Active	13 (77%)

* Pacific islander = Micronesian.

** European derived = White, Portuguese

### Qualitative findings

**Fig 3:**
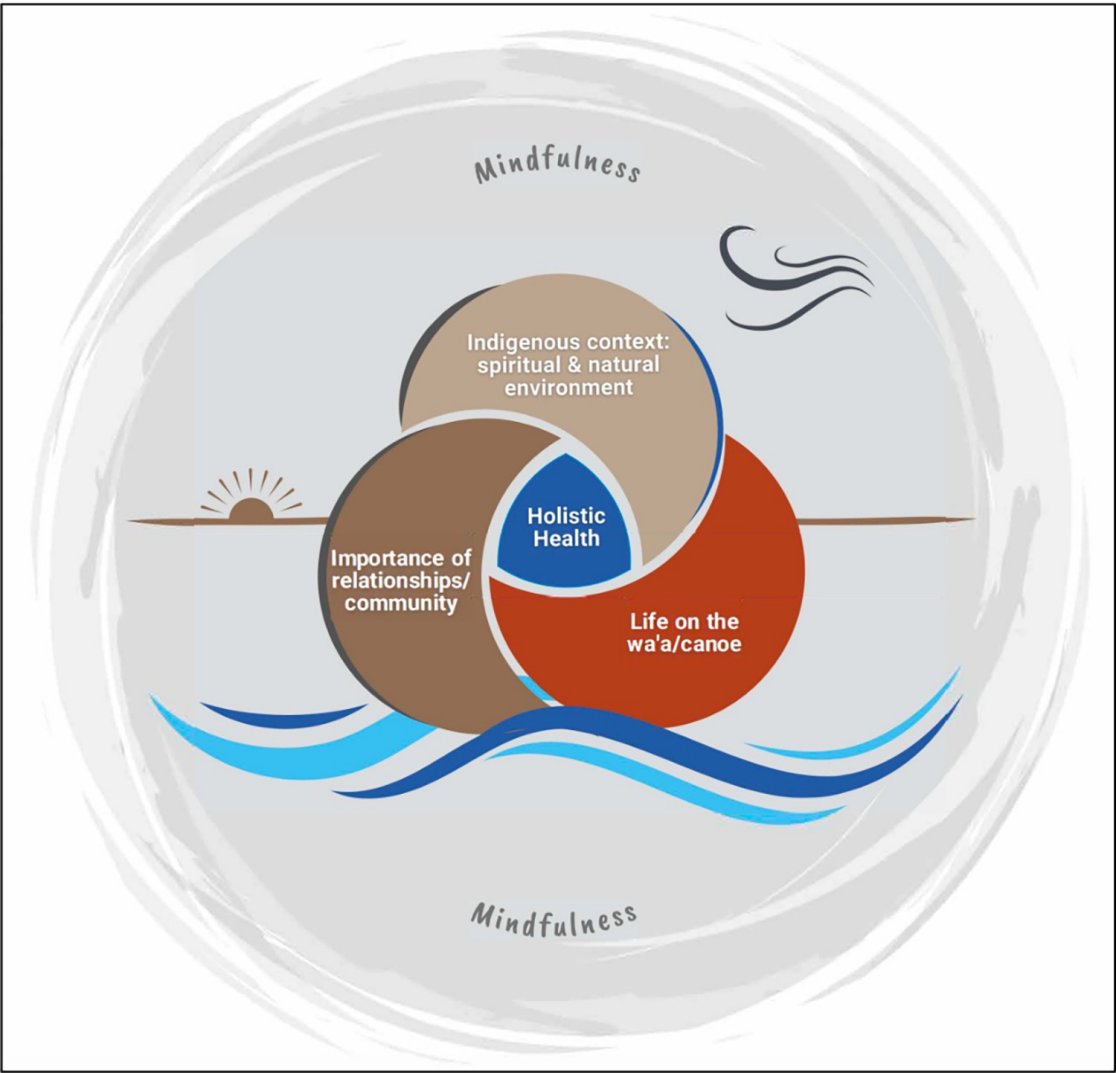
A conceptual model of the inter-relationship of five inductive themes on voyaging and holistic health emerging from experienced voyagers.

Six overarching themes were constructed from the qualitative data, five of which are inductive and one of which (theme #6) is a deductive theme, as the latter encompassed the responses from direct moderator guide questions (**Fig 3**). The six themes include 1) Indigenous context (spiritual and natural environment); 2) Importance of relationships and community (Pilina or relationships); 3) Description of life on the waʻa / canoe; 4) Holistic health; 5) Mindfulness, experiences, and opportunities related to stress reduction and emotional health; and 6) Opportunities for intervention. Below, we provide exemplifying quotations for each of the six themes.

#### Theme #1: Indigenous context (spiritual and natural environment)

The first theme focuses on Indigenous context, including both spiritual and natural environment. Codes that support this theme were centered on discussion about cultural health, cultural spiritual events, mental health and relationship with nature, spiritual focus and health, voyaging values and how to perpetuate these values beyond the voyage itself.

With regards to spiritual health both on and after the voyage, one participant shared:

*Every time I step in a canoe and leave [the dock*], *I’m in the same space as my ancestors*. *Just a different time period*.

Another participant shared their experience with the connection between nature and spiritual and mental health:

*I think to me what helps the most is moving* (*…) in sync with the sunrise and the sunset (…) having a very regular cycle*, *(…) being surrounded by ocean*. *(…) [T]he movement of the canoe is (…) very comforting*… . *I love that feeling*. *(…) [I]t’s those things that just get me out of my brain*. *[Bec]ause normally I’m either thinking of what happened and how I could’ve done it better*, *what’s going to happen and how I’m going to do it better*, *and I’m never right here enough*. *And the canoe makes you (…) you’re constantly just right here now*.

Similarly, another participant elaborated on the spiritual relationship between nature and island people:

*There’s no outside influences*, (*…) so (…) you have to listen*, *you have to see your environment around you*, *because your life depends on it*. *I remember being with [a non-Polynesian crewmember] in Sāmoa and she (…) asked*, *“[H]ow come we go to so many churches [when we stop at ports]*?*” (…) We talked about it*, *and it was because Island people think that way*. *You have to have a higher something*, *because [your] environment depends on the higher being*.

Participants discussed feelings of connectivity to their ancestors, in a spiritual sense, through the presence of the canoe and the physical act of voyaging, and that the lack of distractions of everyday life (e.g., Internet-accessing devices such as smartphones) create a space for this ancestral connection. One participant shared:

*I mean*, *every time I step onto Hōkū[leʻa] (…) [I] [gasp] fall in love every time*. *(…) She’s just this amazing connection to our past and our future and just the spiritual*… *the mana [spiritual power/ strength] that she has*. *I think it helps my own spiritual health*. *Certain things while you’re voyaging you can’t explain*, *right*? *(…) [A]ll these hoʻāilona [natural phenomena that can be interpreted as cultural signs]*… *I think*, *again*, *being in that space to be able to see things where maybe otherwise you wouldn’t*. *And that feeling of*, *you know*, *feeling closer to our kūpuna [ancestors]*,… *and those that*… *[passed away] not that long ago*, *but aren’t physically with us anymore*…[*we] (…) feel a little closer to them*, *too*.

A key value of Native Hawaiian culture, that of oneness and connectivity, was clear in many discussions, as demonstrated in this quotation:

*Over time*, *we have been learning that we’re all different*, *but we have to have the same vision to get somewhere*. *He waʻa he moku; he moku he waʻa* [*The canoe is our island; the island is our canoe] isn’t a metaphor anymore*. *Expectations of self and others are the same now*.

#### Theme #2: Importance of relationships and community (Pilina)

The second theme revealed the importance of relationships and community (Pilina) within voyaging networks. Codes that clustered to support this theme include that of communication, interdependence and familial feelings among crew members, emotional connectivity, and mental health specific to tolerance and patience. The relationships discussed include those among crew members, crew leadership, family who was not on the voyage, as well as the community at home and at visited port communities.

One crew member shared about the importance of relationships and voyaging:

*[NAME]*, *when he’s captain*, *before you leave a port*, *always in a circle you know*, *[for] a pule [prayer]*, *he always says*, *“We leave here as acquaintances or as friends*, *but we always come back as family*.*” And I think he inserts that familial relationship (…)*, *how important that is*, *that we all work together*, *get along*, *and it represents the success of our voyage*. *(*…*) [W]e all have our contributions that contribute to that success*, *and it takes* *all* *of us*. *And in the first few pages of our crew manual it says*, *“I am part of something great*.*” (…) [W]hen I first read that as a first-time crew member*, *I really took that to heart*, *because I felt privileged*. *You feel (…)*, *“Wow*, *I got selected to do this*,*” and then you want to do all that you can do*, *to just make up for (…) why you got selected* (*…)*. *[I]f you believe that [you are part of something great]*, *then you’re going to give all a 100 plus of yourself to contribute to that success*, *whatever project or voyage (…)*.

A voyaging leader discussed their observations of relationships among voyaging crews:

*I’ve done a lot of sailing*. *(…) [T]he reason I [voyage now] is the interaction between the crew*. *It just absolutely fascinates me (…) how you can take people from all walks of life and put them on this incredibly small platform and say*, *“Okay*, *we’re going to have to be dependent on each other*. *You have no choice*.*” All egos are left at the dock*, *for the most part*, *without being told to have to do that*. (*…) [O]nce a major voyage [2,000 miles or more] is done*, *(…) those people become ʻohana [family]*. *You never lose that*.

Participants discussed the deep trust and reliance on one another that is fostered during voyages, as discussed here:

*That’s part of this tight bond that we have*, *because we trust each other with our lives*, *and that camaraderie is developed over time*, *and especially on the voyage*. *When you come back*, *you’re like brothers and sisters; you trust each other no matter what*. *You’ll do anything for each other*… .

Crew members admitted that voyaging relationships are unique and not often replicated outside of the voyaging community, with its cultural basis of interconnectivity, as shared here:

*In this discussion*, *you can hear the sense of community that comes out*, *about how people are concerned for either each other*, *or the canoe*, *or their watch [work groups and their shifts on a voyage]*, *or these different elements that make up really any community*. *(…) And that’s a refreshing thing*, *because I think at home*, *there’s so much chaff that gets stuck in the middle of it that you don’t necessarily have that kind of opportunity to reflect and really think about other people*. (*…) And I think that’s indicative of the old way that things were*, *the old sense of community*, *and that some of us maybe seek out the canoe because we know that that exists there*, *and it doesn’t exist everywhere else for us*.

#### Theme #3: Description of life on the waʻa / canoe

The third theme is that of description of life on the canoe (waʻa). Participants discussed the physical space of the canoe as well as the implications of this physical space and its distance from land, regarding food and water intake, mental focus, voyaging values, and descriptions of both the canoe itself and roles on the canoe. In general, participants indicated the canoe had a positive reputation for transformation and connectedness.

One crew member participant shared how the canoe and the environment can “recalibrate” oneself:

*I think the canoe really helps*. *It always recalibrates your moral compass*. *You* (*…) realize how precious things are in life*, *whether it’s a human being or the marine life we look at*, *the water we’re on*, *the air we breathe*, *how critical all that stuff is*. *And how important it is for society and people to work together*, *to resolve common problems and challenges*.

Another voyaging leader explained the logistics of the canoe and how the lack of a compass or global positioning system (GPS) on a canoe has implications beyond navigation:

*Sailing on these canoes*, *thousands of miles out on the ocean*, *it’s pretty outside the box in this day and age*. *(…) [W]hen people who sail on other canoes that use GPS all the time (…) come sail with us*, *and there’s no compass and they’re just watching the navigators work* (*…)*, *it really blows their mind*. *It really does*. *They go*, *“These guys really don’t use a compass; they really use the stars*.*” And I can see them saying stuff like that to others*, *and (…) it does seem like magic*. *But it’s a skillset*.

Crew members discussed the physical implications of living on the canoe, in terms of physical exertion, sleeping arrangements, and roles on the canoe:

*[F]or me physically it was knowing my limits and kind of like how far I can push myself and all*. *Your body goes into somewhat of a shock in the first couple days*. (*…) Whether it be you’re seasick and then you have to kind of get used to that physically*. *But also it’s just not the normal*, *you know; you’re working four hours and you’re off for eight hours*. *And then sometimes you’re working for six hours and you’re off for six hours*, *depending*. *It’s just not your comfy queen-size memory foam mattress*. *And you’re kind of just like hanging off the side of a canoe to use the bathroom*. *It’s not your normal*.

The roles each crew member has on a voyage also have implications for fostering togetherness, especially in dangerous or emergent situations:

*[Voyaging is] such an intense thing that the people you’re with*, *they’re yours forever (…) especially when you’re in high stress*, *[a] storm*, *somebody goes overboard*, *something happens*, *medical conditions* (*…) where it’s like*, *“No*, *we’re going to stick together; we’re going to figure this out*. *Everybody gets to go home*.*”*

#### Theme #4: Holistic health

This theme encompasses the different facets of health discussed by crew members and leaders. Codes supporting this theme include physical health (e.g., weight changes, healthy food, sleep, and water intake), mental or emotional health (e.g., clarity, tolerance), and spiritual health (e.g., focus, connection). Participants suggested they were physically stronger, had more focus and clarity, and had improved sleep–and that importantly, these health benefits often impacted their sense of belonging and peace.

Often, effects lasted beyond the end of any given voyage, as exemplified here:

*From the first time I was on the canoe*, *I melted into the space*. *I was like*, *“This is where I belong*.*” (*…*) [S]o*, *I looked forward to being on this last voyage*, *because I wanted my heart to stop bouncing all over the place*. *(*…*) And that calming effect lasted months*. *I don’t recall how long*, *but it was quite a while*. *And on that last voyage*, *there [were] two other (…) voyagers that have heart arrhythmias*. *And they stayed on their meds*, *but I never took meds in the first place*. [*Voyaging] made a big difference [for me]*.

One participant shared that voyaging was the healthiest part of his life:

*[W]hen voyaging was almost a daily part of my life*, *it was some of the most healthy times in my life*. *(…) Voyaging was a lifestyle*. *A daily lifestyle*. *(…) [O]nce you go on a voyage that’s at least three weeks long*, *I would see my body start changing*, *adapt[ing] better*. *There was some learning curve for my body*, *but I would get to the point where I don’t need high blood pressure medicine*. *You don’t have upset stomach because you get into a rhythm*, *of*, *I guess what you’re eating and constant moving on the canoe*, *with your core*. *[Once voyaging was no longer a regular part of the crewmember’s life*:*] [E]very time I went back to the canoe*, *I would realize that health improved*. *(…*) *[G]etting on the ocean brings back not just the physical health*, *but sanity*.

Participants understood the importance of improved holistic health for their whole community, as evidenced here, in quotes from two experienced voyagers:

*Speaker 1*: *(*…*) [I]f you’re talking about health*, *in the broad aspects of health*, *and how we look at [the] health of native communities*, *and how those native communities are tied to place*, *and they’re tied to practice*, *and they’re tied to each other*, *that really what we are and have embarked upon for the last 40-something years*, *is the regeneration of this practice that obviously was well thought out and well-funded through resources in terms of*, *not dollars*, *but in terms of the community supporting it for a thousand years (…*). *And what we are re-finding are the missing pieces of what we forgot*… . *[A]s we are doing it and figuring it out*, *we are inherently becoming more healthy at being us*, *from the native perspective of being us*.

Speaker 2: How (…) we [describe] health [being] impacted by that collective experience.

#### Theme #5: Mindfulness, experiences, and opportunities related to self-awareness, stress reduction and emotional health

While closely related to theme #4 (holistic health), the theme of mindfulness and the experiences and opportunities of it related to stress reduction and emotional health and wellbeing are distinct because of the specific emphasis given to them and the frequency they were discussed. Codes that comprise this theme include gratitude, disconnection from “regular” life, mental focus and health (e.g., tolerance), respect for nature, self-awareness, appreciation of simplicity, and transformative experiences that include changes in attitude and perspectives, life changing shifts.

One participant elaborated on “how the canoe ‘inspires one to be a better human’”:

*I feel like emotionally and intellectually*, *that depth*, *that opportunity to just really be introspective and reflective* (…*)*. *[W]hen you’re in society in your daily life*, *it’s kind of like your cup has fallen over*, *and so your time*, *and especially your energy*, *is just this thin film of water on the table and you’re stretching it out as far as it will possibly go*. *And then being on a canoe is this opportunity to kind of upright your glass*, *and you get filled up with water that allows you to channel the energy and just kind of go deep*.

Participants shared how they could apply the newfound sense of mindfulness to life beyond the canoe. For example, by disconnecting together, crew members created a community mindfulness that is more impactful because the focus and purpose are shared:

[*W]hile that can be maddening*, *it’s also calming*, *because you can take that experience and start to apply it to this greater space that is Hawaiʻi*, *that is the world*. *And really just kind of appreciate people and understand and just be more empathic too*. *It’s a very powerful thing*.

Another participant suggested how the canoe allowed them to reconnect with the inner strength and peace that was already inside of them:

*I think it’s always been in you*. *And it’s life changing because you figured out that you can reach it*. *And you can actually hold it and appreciate it*, *and even more understand it*… *where it doesn’t just go away and pass you by* (*…)*. *[Y]ou’re in a situation where it has an opportunity to really show itself and express itself*, *and [you] can be comfortable with that sort of gut feeling or inner strength or soul*. *And that makes it life changing*, *because it’s so focused when you’re tied in with [it]*.

#### Theme #6 (deductive theme): Opportunities for intervention

This was a theme generated directly from a moderator guide question prompting participants to talk about how their voyaging experiences might be applicable for a broader audience. Their responses included: suggestions for improved mental and physical health, the importance of training for a voyage (e.g., preparing family and work, mental preparation, honing the logistics of sailing skills) and the need for support for transition from ocean voyaging back to land.

Crew member and voyaging leadership participants largely agreed with the potential benefits of voyaging for broader audiences on land, as demonstrated here:

*That’s the whole thing of when we teach the kids or even just people when they ask*, *and it is how we live on a canoe is how we should live on land*. *So caring*… *that whole thing I think does carry over with all of us*. *We want to live that same way (*…*) more simpl[y] and reuse everything [because] our resources are limited*. *And I think we all try to carry* (*…) [h]ow we live on the canoes [to] how we should live on land*.

Another participant shared how the values emphasized in voyager training are applicable and beneficial far beyond voyaging culture, as indicated here:

*[A] lot of [voyaging training is about] letting go of your individualism*. *That it’s not about you*. *You’re not the most important thing*. *And it’s easy to see people who are like that*. *And really*, *to be a voyager is to be a community person and not individualistic*, *[such as]*, *“I’ve got to get this*, *I’ve got to get the best grade*, *I’ve got to get the fastest time*, *I’ve got to get*… *me*, *me*, *me*, *me* (*…)*.*” And that’s not what we’re looking for*.

Some shared the importance of training and preparation prior to a voyage and pointed out that others who were not intending to go on a voyage could participate in this preparation period and receive the benefits from these activities. One shared their experience training for a voyage:

*I went to Halapē*, *which is off of Volcanoes National Park*, *before I went voyaging*. *It was really important for me to do that*, *because there’s switchbacks*, *and it’s lava miles versus real miles*. *And you’re in the middle of nowhere*, *with nothing for days and your heavy pack*. *And really there’s no vegetation throughout a lot of it*. *Where you’re with your friends*, *you have to be like*, *“Okay*, *face Kona*, *I’m gonna use the bathroom*.*” But having that*, *in such a short amount of time*, *and the people that I camped with*, *really creates a voyaging scenario*. *You have that sense of closeness*, (*…) and you have to fish and depend on others; it’s such a good scenario to mimic something like [voyaging]*.

Participants discussed that being in a “team” without many resources, “out in nature”, and with a shared goal that requires team-based critical thinking could “replicate” some of the benefits of voyaging, as suggested here:

*[N]ot having many resources*, *something that feels like everyone’s equal*, *you’re not bringing egos*, *there’s no ranking or something*, *where (…) the goal is the same for everyone*. (*…) That could help*.

One key opportunity for intervention included support for voyagers who are transitioning to return to land after any given voyage, because many suggested this was a distinct area of struggle, as exemplified here:

*I think* [*we] could do more in preparing people for the transition*. *(…) [T]here’s a transition*…*of leaving everything back home and having to switch your family off so that you can focus on your new family*. *(*…*) [W]hen you come home*, *(…) you switch off your canoe family that you just been in hell and high water with to go back to your regular family*. *And I think [we] could do more to talk about the end part of when you come home*. *I’ve seen crew members that go down hard and disappear for weeks in a depression*, *because they can’t make the switch*.

## Discussion

Overall, five (5) major inductive themes emerged which generated a visual representation of the interrelationship among them. Three of these themes served as anchoring constructs that were strongly endorsed and expressed as being foundational and closely interrelated: #1) Indigenous context: spiritual and natural environment; #2) Importance of relationships and community; and #3) Life on the waʻa/canoe. Collectively, these three themes were interwoven with each other and integral to theme #4) Holistic health. Holistic health was described as multi-faceted, encompassing physical, mental and spiritual health, among others. The #5) theme, Mindfulness, focused on the interrelationship of the four other inductive themes and served as a guide to and awareness of them (**Fig 3**). The mindfulness theme expressed a need to be present in the moment, focused and attentive to the fully immersive experience of voyaging, including crew, the natural environment, the waʻa, oneself, and the ever-present ancestral spirits. Many of the experienced voyagers were keenly aware of the sacred space the waʻa (canoe) created, referencing all five inductive themes as being interrelated towards striving for a better version of themselves. This sense of completeness and holistic wellbeing was conveyed as life-changing, ancestrally profound and often extending beyond their physical time on the waʻa (canoe).

The single deductive theme generated salient recommendations drawn from years of collective experience during the WWV and from voyages prior and since. Many participants suggested that more could be done to support families, communities, and the voyagers themselves to cope and understand the process of transitioning back to their daily lives with their ʻohana (family), work, and prior routines. Teaching others core thematic values of relationships, caring, humility, and indigenous or ancestral knowledge was cited as a potential approach that was recommended to be implemented in a community context, beyond voyaging. Insightfully, there was strong agreement on the idea that voyaging was not about or for any single individual. Rather, voyaging is about and for the collective group and the existence of a “voyaging lifestyle”.

This particular group of participants was diverse in age, gender, educational attainment and racial/ethnic background, but all of these demographics were set aside when a voyage was underway, and crew were interdependent on each other. The whole voyaging crew working in synchrony was more effective and powerful than the impact of any single person. This interweaving of Indigenous context, relationships and community with the rhythm of life on a canoe forged a state of holistic health (mental, physical, spiritual, emotional) in which mindfulness was essential and encompassing of all themes. Our final deductive theme provided specific strategies that these experienced voyagers would recommend as potential opportunities for future training, support and preparation that extend beyond oceanic voyaging, including on land and within the broader community.

### Results in context

The results of this study highlight a growing recognition of how practicing one’s traditional culture has been linked to holistic health benefits. In our study, highly experienced cultural practitioners reported on the profound impact of Polynesian oceanic voyaging as a fundamental act of practicing one’s ancestral knowledge and skills. These “expert” voyagers found a deeper understanding of relationships, environmental influences (natural and spiritual), and the sacredness of space on a traditional voyaging canoe. Collectively these integrated themes promoted holistic health and mindfulness in a unique indigenous context, transforming the ancestral practice of ocean voyaging back into a way life. These results support our previous study, confirming the transformative influence of ocean voyaging on health and wellbeing through the lens of medical doctors who were “novice” voyagers and in a more limited capacity, during the WWV [41]. Interestingly, both medical and experienced voyagers expressed the overall positive impact of the voyaging experience on human health and wellbeing that extended beyond the actual physical time spent on the canoe. In summary, implementing traditional cultural practices (i.e. voyaging) enabled holistic health and wellbeing, which encompassed more than the absence of disease but rather became a constellation of place-based, value-focused themes, reaching into spiritual, environmental (i.e. the theme of “indigenous context”), and relational constructs, with a priority for the collective relationship with and of community (i.e the theme of “relationships and community”) [55]. The theme of “life on the waʻa” defines a sense of place that is intimate and sacred [37,56] (**Fig 3**).

Our study results strongly support the growing recognition that cultural activities, such as Polynesian oceanic voyaging, provide unique opportunities to positively impact individuals across multiple dimensions of health and wellbeing. To put our findings into context of other indigenous cultures, a study, among the Yellowknife Dene First Nations People in Canada, found that “cultural camps” attended by indigenous youth reported that being physically active is to be culturally active, meaning that physical activity strengthened the youths’ cultural identity and by extension the community’s indigenous identity [citation 57]. Furthermore, the study defined cultural identity by five themes: 1) respecting elders (i.e. bearers of knowledge); 2) passing on knowledge (i.e. generational); 3) inclusiveness (i.e. of a collective group); 4) land (i.e. natural environment as a cultural place); 5) traditional practices (i.e. cultural practices as a way of life) [57]. Similarly, our study found the importance of the natural and spiritual environment and of relationships, inclusive of community, as major themes. Moreover, the authors of the Yellowknife Dene study report on the indigenous perspective that physical activity is not exercise but truly “a way of life”. That is, an indigenous worldview that practicing one’s cultural through physical participation is, in reality, a traditional way of life that manifests as holistic health and wellbeing [57]. Multiple studies have been published describing active participation in traditional practices (i.e. paddling, hunting, dancing, etc.) as beneficial to emotional, mental, spiritual, and physical health while reducing the presence or onset of disease [58–61].

The construct of “place-based” as a fundamental feature of traditional ecological knowledge is also well recognized in the published literature and supported by our results [11,12,41,56,62]. While many indigenous people were physically removed from their ancestral lands, the existing literature and our current study emphasizes the importance of connection to place (i.e. land, ocean, etc.) as a critically important feature of practicing one’s culture. Our results suggest that being immersed in the spiritual environment within nature provides the proper context for achieving holistic health through cultural knowledge and practice. For example, by being mindful of the expansiveness and constantly changing power of nature while at sea, the experienced voyagers found a reciprocal rhythm with their environment and each other, enabling them to replicate success, using a NH foundation and cultural approach often referred to as an “ancestral knowledge systems” [62–64]. Equally, the context and sense of place on the isolated and small area of the waʻa kaulua (double-hulled canoe) served to challenge the individuality of the crew and enhance the cultural practice and need for tolerance, mutual reliance, community, trust and respect.

Our study also outlines future potential of cultural-based physical activities that may offer multiple health benefits that extend beyond fitness, weight loss, muscle strength or any other physical measurement. Ocean voyaging seemed to be a positive influence on multiple levels such as emotional, mental and spiritual wellbeing. Its emphasis on relationships, particularly community relationships, is also supported by studies of other indigenous populations with a collectivist perspective [57,58,65]. Our conceptual model resonates with other indigenous models that integrate intra-personal, inter-personal, family/community, systems/environment and cosmos/mother earth into the fundamentals of relationships [58]. In essence, our study parallels the majority of indigenous literature which is strength-based, collectivist and holistic in its worldview and contextual [57,59,65].

In comparison with European-derived perspectives, we initially considered the physicality of ocean voyaging [41], as a major health factor. However, based on our results from novice medical voyagers as well as highly experienced voyagers, we found that oceanic voyaging provided much more than physical exertion, including reports of residual positive effect after the voyage had ended [41]. Few studies have examined how cultural practices and traditional knowledge have sustained indigenous peoples for millennia. Yet a growing movement toward re-vitalizing indigenous knowledge of nutritional practices, farming and land-ocean management is gaining in popularity [66,67].

Other examples of re-enacted significant cultural events contributing to the health of multiple generations of indigenous people are consistent with our overall findings. Lewis et al. [65] reported on American Indians residing in Oklahoma and the importance of a shared cultural context as being effective in promoting health benefits during an intensive cultural-based activity known as the “Remember the Removal” (RTR) program. The RTR program retraces the “Trail of Tears”, a forced 1,000-mile march of American Indian families removed from their ancestral homelands [65,68]. The authors reported on physical, mental, and dietary changes, as well as cultural learning, resilience and traditional values that were significantly improved following the completion of the RTR program [65].

In parallel, our results also found holistic health benefits by retracing the ancestral practice of voyaging, wayfinding and communal relationships. Our studies provide further evidence of how cultural practices, knowledge and values can be implemented within a cultural context to address the multiple layers and complexities of health disparities experienced by indigenous populations in the USA and the Pacific [13,31]. The potential for oceanic voyaging and other traditional cultural activities to offer relevant and practical strategies for promoting holistic health and wellbeing and is key to reversing longstanding health inequities that emerged as a result of European/American-derived colonization.

The key implications of the findings of our study are that potential solutions for achieving holistic health and wellbeing in contemporary times may stem from our capacity to practice and engage in our ancestral knowledge systems and the enactment of cultural practices, traditions, and values. This study supports the idea that Indigenous knowledge, when applied to today’s environment and human challenges, holds promise for reversing some of the most persistent health disparities impacting the wellbeing of Indigenous populations such as NHs and PIs [36].

What remains unclear is the precise mechanism of how ocean voyaging invokes its health benefits at a biomedical and cellular level. A growing area of research has recently proposed the influence of immuno-epigenomics and the gut microbiome axis as key to reversing health inequities among NHs [29,30]. Further investigations are needed to examine the impact of culture as a tool to improve health and wellbeing in a sustainable, community-based and accessible way, within contemporary settings and working respectfully with the culture itself [29,30].

### Strengths and limitations

A major strength of this study was the use of rigorous methods and a diverse team of co-authors who provided a rich environment in which to explore the perspectives and insights of a demographically diverse cohort of participants (e.g., varied age range, sex, education). The analytical approach was further strengthened by member checking and triangulation of data, which informed the final analysis and study findings. This paper adds to the nascent yet growing literature on Indigenous knowledge and practices, which are core to addressing a broad range of health inequities among Indigenous and other marginalized populations. Our results also discuss opportunities to design future programs and serve as a foundation for future interventions to reverse persistent and complex health disparities that have been difficult to overcome in the past. We acknowledge a limitation inherent to a small sample size, yet as with all qualitative studies, these findings are not meant to be generalizable beyond this sample. Further, we were successful in reaching thematic saturation, and the findings were affirmed by member checking. Future studies are needed to experimentally test possible implementation studies and to include a broader diverse population (generalizability), while at the same time considering the inclusion of ocean-based and land-based environmental contexts. Further investigation into understanding the underlying biology and mechanism of action stimulated by this preliminary data is yet another strength to confirm the connection of culturally grounded activities as potential solutions for contemporary health risks for a wide range of chronic health conditions.

## Conclusions

Traditional ocean voyaging was strongly endorsed by highly experienced voyagers as a major contributor to achieving holistic health and wellbeing. Core constructs include: 1) an Indigenous context of spiritual and natural environment; 2) relationships, with emphasis on community; and 3) life on the waʻa (canoe) that enabled clarity, focus and a sense of place. All three constructs were integral to holistic health (4th theme) and mindfulness (5th theme), which raised focused awareness, reduced stress and enhanced emotional health during voyaging, enabling life-changing experiences that were unique and extended beyond the physical voyage itself. Future studies on implementing ocean voyaging as a cultural practice to achieve health are warranted and may include recommendations from these experienced voyagers to improve community health and wellbeing. In summary, culturally grounded practices such as traditional Polynesian oceanic voyaging have been found to have a significant role in achieving holistic health and, thus, hold promise for reversing health inequities in Native Hawaiians, Pacific Islanders and other global indigenous populations at similar risk.

## Supporting information

S1 File Moderators guide. (PDF)

S2 File Sociodemographic questionnaire. (PDF)

## References

[1] Sear DA, Allen MS, Hassall JD, Maloney AE, Langdon PG, Morrison AE, et al. Human settlement of East Polynesia earlier, incremental, and coincident with prolonged South Pacific drought. Proc Natl Acad Sci [Internet]. 2020 Dec 10;117(16):8813–9. Available from: https://www.pnas.org/content/117/16/8813 doi: 10.1073/pnas.1920975117 32253300 PMC7183181

[2] Kirch P V. On the Road of the Winds: An Archaeological History of the Pacific Islands Before European Contact [Internet]. ACLS Humanities E-Book. University of California Press; 2002. Available from: https://books.google.com/books?id=H7IwDwAAQBAJ

[3] Lewis D., We the Navigators: The Ancient Art of Landfinding in the Pacific [Internet]. University of Hawaii Press; 1994. Available from: https://books.google.com/books?id=SiCCMB6xQJoC

[4] Crowe A. Pathway of the Birds: The voyaging achievements of Māori and their Polynesian ancestors. Honolulu: University of Hawaiʻi Press; 2018. 288 p.

[5] Thompson SL, Chenhall RD, Brimblecombe JK. Indigenous perspectives on active living in remote Australia: a qualitative exploration of the socio-cultural link between health, the environment and economics. BMC Public Health [Internet]. 2013 Nov 15;13(1):473. Available from: http://bmcpublichealth.biomedcentral.com/articles/10.1186/1471-2458-13-473 10.1186/1471-2458-13-473 PMC3662620 23672247

[6] Athens JS, Rieth TM, Dye TS. A Paleoenvironmental and Archaeological Model-Based Age Estimate for the Colonization of Hawai’i. Am Antiq. 2014;79(1):144–55.

[7] Kim SK, Gignoux CR, Wall JD, Lum-Jones A, Wang H, Haiman CA, et al. Population Genetic Structure and Origins of Native Hawaiians in the Multiethnic Cohort Study. PLoS One. 2012;7(11):1–10. doi: 10.1371/journal.pone.0047881 PMC3492381 23144833

[8] Kirch P. When Did the Polynesians Settle Hawai’i? A Review of 150 Years of Scholarly Inquiry and a Tentative Answer. Hawaiian Archaeol. 2011 Jan 1;12.

[9] Thompson C. Sea people: the puzzle of Polynesia. New York, NY: Harper, an imprint of HarperCollinsPublishers; 2019.

[10] Holmes T. The Hawaiian Canoe [Internet]. Editions Limited; 1993. Available from: https://books.google.com/books?id=WzMHAAAACAAJ

[11] Finney BR, Among M, Babayan C, Rhodes R, Crouch T, Frost P, et al. Voyage of Rediscovery: A Cultural Odyssey Through Polynesia [Internet]. University of California Press; 1994. Available from: https://books.google.com/books?id=1eElDQAAQBAJ

[12] Finney BR. Sailing in the Wake of the Ancestors: Reviving Polynesian Voyaging [Internet]. Bishop Museum Press; 2003. 192 p. Available from: https://books.google.com/books?id=V7btAAAAMAAJ

[13] Paglinawan LK, Paglinawan RL, Kauahi D, Kanuha VK. Nana I Ke Kumu, Helu Ekolu. First. Vol. 3. Honolulu, Hawaiʻi: Liliuokalani Trust; 2020. 136 p.

[14] Harden MJ. Voices of Wisdom: Hawaiian Elders Speak. Kula, Hawaiʻi: Aka Press; 1999. 240 p.

[15] MacKenzie MK. Historical Background. In: Native Hawaiian law: a treatise. Honolulu, Hawaiʻi: Kamehameha Publishing; 2015. p. 5–74.

[16] Low S. Hawaiki Rising: Hōkūle‘a, Nainoa Thompson, and the Hawaiian Renaissance [Internet]. 1st ed. University of Hawaii Press; 2019. Available from: https://books.google.com/books?id=olvGDwAAQBAJ

[17] Blaisdell RK. The Impact of Disease on Hawaiʻi’s History. Hawaiʻi Med J [Internet]. 2001;60(11):295–6. Available from: http://files/460/Blaisdell_Impact of Disease on Hawaiʻi’s History.pdf 11797498

[18] Blaisdell K. I Hea Nā Kānaka Maoli? Whither the Hawaiians? Hūlili Mult Res Hawaiian Well-Being [Internet]. 2005;2(1):9–18. Available from: http://files/459/Hulili_Vol2_2.pdf

[19] Mau MK, Sinclair K, Saito EP, Baumhofer KN, Kaholokula JK. Cardiometabolic Health Disparities in Native Hawaiians and Other Pacific Islanders. Epidemiol Rev [Internet]. 2009 Dec 10;31(1):113–29. Available from: doi: 10.1093/ajerev/mxp004 PMC2893232 19531765

[20] Qato DM. Reflections on “Decolonizing” Big Data in Global Health. Ann Glob Heal. 2022;88(1):56. doi: 10.5334/aogh.3709 35936229 PMC9306674

[21] Kanaya AM, Hsing AW, Panapasa S V, Kandula NR, Araneta MRG, Shimbo D, et al. Knowledge Gaps, Challenges, and Opportunities in Health and Prevention Research for Asian Americans, Native Hawaiians, and Pacific Islanders: A Report From the 2021 National Institutes of Health Workshop. Vol. 175, Annals of internal medicine. United States; 2022. p. 574–89. doi: 10.7326/M21-3729 34978851 PMC9018596

[22] Panapasa S V, Mau MK, Williams DR, McNally JW. Mortality Patterns of Native Hawaiians Across Their Lifespan: 1990–2000. Am J Public Health [Internet]. 2010 Dec 10;100(11):2304–10. Available from: https://ajph.aphapublications.org/doi/full/10.2105/AJPH.2009.183541 20864716 10.2105/AJPH.2009.183541 PMC2951954

[23] Mau MKLM West MR, Shara NM Efird JT, Alimineti K, Saito E, et al. Epidemiologic and clinical factors associated with Chronic Kidney Disease among Asian Americans and Native Hawaiians. Ethn Health [Internet]. 2007 Dec 10;12(2):111–27. Available from: doi: 10.1080/13557850601081720 17364897

[24] Nakagawa K, Koenig MA, Seto TB, Asai SM, Chang CW. Racial disparities among Native Hawaiians and Pacific Islanders with intracerebral hemorrhage. Neurology [Internet]. 2012 Dec 10;79(7):675–80. Available from: https://n.neurology.org/content/79/7/675 doi: 10.1212/WNL.0b013e3182608c6f 22815551 PMC3414664

[25] Braun L, Mokuau K, Tsark U. Cultural Themes in Health, Illness, and Rehabilitation for Native Hawaiians: Observations of Rehabilitation Staff and Physicians. Top Geriatr Rehabil. 1997;12(3):19–37.

[26] Grandinetti A, Chang HK, Chen R, Fujimoto WY, Rodriguez BL, Curb JD. Prevalence of overweight and central adiposity is associated with percentage of indigenous ancestry among native Hawaiians. Int J Obes [Internet]. 1999 Dec 10;23(7):733–7. Available from: https://www.nature.com/articles/0800921 doi: 10.1038/sj.ijo.0800921 10454107

[27] Albright C, Mau M, Choy L, Mabellos T. Physical Activity among Native Hawaiians and Pacific Islanders. In: Physical Activity in Diverse Populations: Evidence and Practice. Taylor & Francis; 2017. p. 123–42.

[28] Look MA, Soong S, Kaholokula JK. Assessment and Priorities for Health and Well-Being in Native Hawaiians and Pacific Islanders [Internet]. 2020. Available from: http://rgdoi.net/10.13140/RG.2.2.22162.89286

[29] Dye CK, Corley MJ, Ing C, Lum-Jones A, Li D, Mau MKLM, et al. Shifts in the immunoepigenomic landscape of monocytes in response to a diabetes-specific social support intervention: a pilot study among Native Hawaiian adults with diabetes. Clin Epigenetics [Internet]. 2022;14(1):1–21. Available from: 10.1186/s13148-022-01307-6 35851422 PMC9295496

[30] Wells RK, Kunihiro BP, Phankitnirundorn K, Peres R, McCracken TA, Umeda L, et al. Gut microbial indicators of metabolic health underlie age-related differences in obesity and diabetes risk among Native Hawaiians and Pacific Islanders. Front Cell Infect Microbiol. 2022;12(December):1–17. doi: 10.3389/fcimb.2022.1035641 36619744 PMC9812644

[31] Makuau N, Crabbe K, Fox K. Kū ka ʻŌhiʻa i ka ʻAʻā—ʻŌhiʻa That Stands amid the Lava Fields. Hūlili Multidiscip Res Hawaiian Well-being [Internet]. 2019;11(2):323–38. Available from: http://files/446/Hulili_Vol11.2_Mokuau.pdf

[32] Walters KL, Johnson-Jennings M, Stroud S, Rasmus S, Charles B, John S, et al. Growing from Our Roots: Strategies for Developing Culturally Grounded Health-Promotion Interventions in American Indian, Alaska Native, and Native Hawaiian Communities. Prev Sci [Internet]. 2018 May 25; doi: 10.1007/s11121-018-0952-z Available from: https://www.ncbi.nlm.nih.gov/pmc/articles/PMC6502697/ PMC6502697 30397737

[33] Kaholokula JK, Look M, Mabellos T, Zhang G, de Silva M, Yoshimura S, et al. Cultural dance program improves hypertension management for native Hawaiians and Pacific Islanders: A pilot randomized trial. J Racial Ethn Heal Disparities [Internet]. 2015;4(1):35–46. Available from: doi: 10.1007/s40615-015-0198-4 27294768 PMC5283501

[34] Kaholokula JK, Wilson RE, Townsend CKM, Zhang GX, Chen J, Yoshimura SR, et al. Translating the Diabetes Prevention Program in Native Hawaiian and Pacific Islander communities: the PILI ‘Ohana Project. Transl Behav Med [Internet]. 2014 Dec 10;4(2):149–59. Available from: doi: 10.1007/s13142-013-0244-x 24904698 PMC4041922

[35] Kanaʻiaupuni SM. We Voyage for the Earth: Cultural Advantage as a Global Education Framework. In: McKinley EA, Smith LT, editors. Handbook of Indigenous Education [Internet]. Singapore: Springer Singapore; 2018. p. 1–27. Available from: 10.1007/978-981-10-1839-8_6-1

[36] Ingersoll KA. Waves of Knowing: A Seascape Epistemology [Internet]. Duke University Press; 2016. 160 p. Available from: https://books.google.com/books?id=gfI0DQAAQBAJ

[37] Kikiloi K. Rebirth of an archipelago: sustaining a Hawaiian cultural identity for people and homeland. Hūlili Multidisplinary Res Hawaiian Well-Being [Internet]. 2010;6. Available from: http://files/264/Kikiloi - 2010—Rebirth of an archipelago sustaining a Hawaiian c.pdf

[38] McGregor DP. Na Kua`aina: Living Hawaiian Culture [Internet]. University of Hawai’i Press; 2007. Available from: https://www.jstor.org/stable/j.ctt6wr2zc

[39] Guth HK. Protecting and Perpetuating Papahānaumokuākea: Involvement of Native Hawaiians in Governance of Papahānaumokuākea Marine National Monument. In: Governing Ocean Resources: New Challenges and Emerging Regimes: A Tribute to Judge Choon Ho-Park. Leiden: Martinus Nijhoff; 2013.

[40] The Kumulipo: An Hawaiian Creation Myth. 2nd ed. Kentfield, California: Pueo Press; 1997.

[41] Mau MKLM Minami CM, Stotz SA Albright CL, Kana’iaupuni SM Guth HK. Qualitative study on voyaging and health: perspectives and insights from the medical officers during the Worldwide Voyage. BMJ Open [Internet]. 2021 Apr 7;11(7):e048767. Available from: https://bmjopen.bmj.com/content/11/7/e048767 doi: 10.1136/bmjopen-2021-048767 34233995 PMC8264866

[42] Reeves S, Albert M, Kuper A, Hodges BD. Qualitative Research: Why use Theories in Qualitative Research? Br Med J. 2008;337(7670):631–4. 10.1136/bmj.a949 18687730

[43] Lauckner H, Paterson M, Krupa T. Using constructivist case study methodology to understand community development processes: Proposed methodological uuestions to guide the research process. Qual Rep. 2012;17:1–22.

[44] Tracy SJ. Qualitative Quality: Eight “Big-Tent” Criteria for Excellent Qualitative Research. Qual Inq. 2010;16(10):837–51.

[45] Harris JE, Gleason PM, Sheean PM, Boushey C, Beto JA, Bruemmer B. An Introduction to Qualitative Research for Food and Nutrition Professionals. J Am Diet Assoc [Internet]. 2009 Aug 31;109(1):80–90. Available from: https://linkinghub.elsevier.com/retrieve/pii/S0002822308018956 doi: 10.1016/j.jada.2008.10.018 19103326

[46] Rothe JP, Ozegovic D, Carroll LJ. Innovation in qualitative interviews: “Sharing Circles” in a First Nations community. Inj Prev [Internet]. 2009 Dec 23;15(5):334–40. Available from: https://injuryprevention-bmj-com.eres.library.manoa.hawaii.edu/content/15/5/334 doi: 10.1136/ip.2008.021261 19805603

[47] Alfonso DD, Shibuya JY, Frueh BC. Talk‐Story: Perspectives of Children, Parents, and Community Leaders on Community Violence in Rural Hawaii. Public Health Nurs. 2007;24(5):400–8. doi: 10.1111/j.1525-1446.2007.00650.x 17714224

[48] Qu SQ, Dumay J. The qualitative research interview. Qual Res Account Manag [Internet]. 2011 Dec 23;8(3):238–64. Available from: 10.1108/11766091111162070

[49] Ainsworth BE, Jacobs DR, Leon AS. Validity and reliability of self-reported physical activity status: the Lipid Research Clinics questionnaire. Med Sci Sport Exerc [Internet]. 1993 Apr 7;25(1):92. Available from: https://journals.lww.com/acsm-msse/Abstract/1993/01000/Validity_and_reliability_of_self_reported_physical.13.aspx doi: 10.1249/00005768-199301000-00013 8423761

[50] Hsieh H-F, Shannon S. Three approaches to qualitative content analysis. Qual Health Res. 2005;15(9):1277–88. doi: 10.1177/1049732305276687 16204405

[51] Stemler S. An overview of content analysis. Pract Assessment, Res Eval [Internet]. 2000 Aug 4;7(1):17. Available from: https://scholarworks.umass.edu/pare/vol7/iss1/17

[52] Saldana J. The Coding Manual for Qualitative Researchers. 2nd Editio. Thousand Oaks, California: SAGE Publications; 2012.

[53] Hsieh H-F, Shannon SE. Three approaches to qualitative content analysis. Qual Health Res. 2005;15(9):1277–88. doi: 10.1177/1049732305276687 16204405

[54] Paulus T, Lester J, Deptster P. Digital Tools for Qualitative Research. 1st ed. Los Angeles, CA: SAGE Publications; 2014.

[55] Mokuau N, Crabbe K, Fox K. Kū ka ‘Ōhi‘a i ka ‘A‘ā—‘Ōhi‘a That Stands amid the Lava Fields. Hūlili Multidiscip Res Hawaiian Well-Being. 2019;11(2):323–38.

[56] Saffery MLK. Mai Ka Piko a Ke Mole: Clearing Paths and Inspiring Journeys To Fulfill Kuleana Through ʻāina Education a Dissertation Submitted To the Graduate Division of the University of Hawaiʻi At Mānoa in Partial Fulfillment of the Requirements for the Degree of D. 2019;

[57] Tang K, Program CW, Jardine CG. Our Way of Life: Importance of Indigenous Culture and Tradition to Physical Activity Practices. Int J Indig Heal. 2016;11(1):211–27.

[58] Akbar L, Zuk AM, Tsuji LJS. Health and Wellness Impacts of Traditional Physical Activity Experiences on Indigenous Youth: A Systematic Review. Int J Environ Res Public Health. 2020 Nov;17(21). doi: 10.3390/ijerph17218275 PMC7664942 33182405

[59] Masotti P, Dennem J, Bañuelos K, Seneca C, Valerio-Leonce G, Inong CT, et al. The Culture is Prevention Project: measuring cultural connectedness and providing evidence that culture is a social determinant of health for Native Americans. BMC Public Health. 2023;23(1):1–10. 10.1186/s12889-023-15587-x PMC10120477 37085784

[60] Look MA, Maskarinec GG, de Silva M, Seto T, Mau ML, Kaholokula JK. Kumu hula perspectives on health. Hawai’i J Med public Heal a J Asia Pacific Med Public Heal. 2014 Dec;73(12 Suppl 3):21–5. 25535597 PMC4271348

[61] Maskarinec GG, Look M, Tolentino K, Trask-Batti M, Seto T, de Silva M, et al. Patient Perspectives on the Hula Empowering Lifestyle Adaptation Study: Benefits of Dancing Hula for Cardiac Rehabilitation. Health Promot Pract [Internet]. 2014 Mar 27;16(1):109–14. Available from: 10.1177/1524839914527451 24677383 PMC4177511

[62] Huntington H. Using Traditional Ecological Knowledge in Science: Methods and Applications. Ecol Appl. 2000;10(5):1270–4.

[63] Moreno Sandoval CD, Lagunas RM, Montelongo LT, Díaz MJ. Ancestral knowledge systems: A conceptual framework for decolonizing research in social science. AlterNative. 2016;12(1):18–31.

[64] Finn S, Herne M, Castille D. The Value of Traditional Ecological Knowledge for the Environmental Health Sciences and Biomedical Research. Environ Health Perspect. 2017;125(8):1–9. doi: 10.1289/EHP858 28858824 PMC5783664

[65] Lewis M, Myhra L, Smith B, Holcomb S, Erb J, Jimenez T. Tribally specific cultural learning: the Remember the Removal program. Altern An Int J Indig Peoples [Internet]. 2020 Apr 26;16(3):233–47. Available from: http://journals.sagepub.com/doi/10.1177/1177180120952897

[66] Gadamus L. Linkages between human health and ocean health: A participatory climate change vulnerability assessment for marine mammal harvesters. Int J Circumpolar Health. 2013;72(SUPPL.1). 10.3402/ijch.v72i0.20715 PMC3752289 23984268

[67] Schultz R, Abbott T, Yamaguchi J, Cairney S. Indigenous land management as primary health care: Qualitative analysis from the Interplay research project in remote Australia. BMC Health Serv Res. 2018;18(1):1–10. 30541540 10.1186/s12913-018-3764-8 PMC6291963

[68] Johnson-Jennings M, Billiot S, Walters K. Returning to Our Roots: Tribal Health and Wellness through Land-Based Healing. Genealogy. 2020;4(3):91.

