# Supplementary material for: Exploring perspectives and insights of experienced voyagers on human health and Polynesian oceanic voyaging: A qualitative study
Source: PLoS One. 2024 Apr 15;19(4):e0296820. doi: 10.1371/journal.pone.0296820 (PMC11018278; doi:10.1371/journal.pone.0296820)
Supplement: S1 File — (PDF) [file pone.0296820.s001.pdf]

VOYAGING AND HEALTH STUDY

Focus Group & Informant Interview Research Guide

Focus Group Discussion Guide

Consent Process

Consent forms for focus group participants are completed in advance by all those who volunteer to participate. Below is a summary of the information in the consent form that focus group organizers and facilitators should use to make sure participants understand the information in the consent form.

Thank you for agreeing to participate. We are very interested to hear your valuable opinions on the relationship between voyaging and health & wellbeing.

- The purpose of this study is to learn about the perceptions of the crew members who volunteered to serve on the Worldwide Voyage (WWV) about the topic of "voyaging and health and wellbeing".

- The goal of the focus group discussion is to use the collective perspectives and insights of the WWV crew members to help us create and eventually test the effectiveness of a program to improve health and wellbeing outcomes among Native Hawaiian/Pacific Islander (NHPI) adults, and potentially other relevant groups of people, such as elders, adolescents, and children.

- Feel free to discuss your own ideas but also to comment on and agree or disagree with others' comments – we want you to bounce ideas/opinions around the room – there are NO right/wrong answers – and we would like to understand/hear about those issues that seem to make sense to many of you.

- The information you give us is completely confidential, and we will not associate your name with anything you say in the focus group.

- We would like to audio record the focus groups so that we can make sure to capture the thoughts, opinions, and ideas we hear from the group accurately. No names will be attached to the focus groups, and the tapes will be destroyed as soon as they are transcribed. Also, a notetaker will be taking handwritten notes just in case the tape recorder doesn't pick up all the voices, but no names will be associated with his/her notes.

- You may refuse to answer any question and to withdraw from the study at any time.

- We understand how important it is that this information is kept private and confidential. We will ask participants to respect each other's confidentiality.

- If you have any questions now or after you have completed the questionnaire, you can always contact a study team member like me or other identified members of the research team.

VOYAGING AND HEALTH STUDY

Focus Group & Informant Interview Research Guide

Introduction:

1. Welcome

Introduce yourself and other team members including their role (facilitator, moderator, notetaker, etc.). Ask attendees to fill-in the Sign-In Sheet. As is customary in Native Hawaiian gatherings, the research team leader will introduce themselves briefly including their background/interest in this study and to share it in a "talk story" format. Review the following:

- Who we are and what we're trying to do
- What will be done with this information
- Why we asked you to participate

2. Explanation of the process

Ask the group if anyone has participated in a focus group before. Explain that focus groups are being used more often in health research studies, especially among diverse populations such as Native Hawaiians, Pacific Island Peoples (NHPI), Asians, etc. Mention that our research group has used focus groups on multiple occasions to assist with the design and creation of intervention programs based on existing scientific evidence to adapt interventions that are to be used among NHPI and other diverse communities.

About focus groups

- We learn from you (positive, negative, neutral, etc.)
- No "right or wrong" answers. We're gathering information to better understand your experience and perceptions about voyaging and how it has impacted you and those around you with regard to health and well being.
- We're interested in learning from your experience and how it has shaped your thoughts/reflections before and after voyaging on the WWV as well as prior to and since the WWV.
- In this project, we are doing questionnaires, informant interviews and focus group discussions. The reason for using multiple tools is to get more in-depth information from select individuals as well as from smaller groups of people in focus groups. This allows us to understand the context behind the answers given in the written survey and helps us explore topics in more detail than we can obtain using only a single method.

Logistics

- Focus group discussion will last about one to two hours
- Feel free to move around
- Where is the bathroom? Exit?
- Help yourself to refreshments

3. Ground Rules: "Talk-Story" format

In keeping with our local customs, we will be using a "talk story" format that acknowledges the cultural context of the group gathered as well as the place in which the meeting is held. General guidelines will be shared by the group and if not mentioned will emphasize the following:

- Everyone should participate.
- Information provided in the focus group must be kept confidential
- Stay with the group, and please don’t have side conversations
- Turn off cell phones if possible
- Have fun

4. **Turn on Tape Recorder**

5. Ask the group if there are any questions before we get started, and address those questions.

6. Introductions (3-5 min each)

- Go around the table: what you currently "do" (i.e. job, known for..., role on canoe, etc.), where you were born or where your family is from. How long have you been part of the PVS or ‘Ohana Wa‘a?

Discussion begins; make sure to give people time to think before answering the questions and don’t move too quickly. Use the probes to make sure that all issues are addressed, but move on when you feel you are starting to hear repetitive information.

Questions:

Let’s start the discussion by establishing a common understanding of the terms "VOYAGING" and "HEALTH & WELLBEING":

a) VOYAGING: the act of ocean sailing and "living" on our traditional canoes (waʻa kaulua), which are designed for open-ocean sailing and which require the cooperation of other crew members to perform the act.
b) HEALTH & WELLBEING: refers to "holistic health" which is meant to encompass physical, mental, emotional and spiritual health, and therefore indicates more than the absence of disease.

#/Time	Category	Questions	Person
1.	Individual	As WWV crew members, was there any impact of voyaging on your sense of health and wellbeing?<br><br>If so, please describe any changes you may have noticed, why you think these occurred, and how long they lasted.	
		• During or after voyaging, did you notice any changes in your physical health?<br>○ Probes: Describe any changes in weight, fitness, strength or any other physical abilities, including changes in sleep patterns.<br>○ If you are comfortable sharing, can you tell us about any non-voyaging-related medications that you needed to maintain while voyaging? → If so, were there any changes in your need for that medication?<br>• During or after voyaging, did you notice any changes in your mental health?<br>○ Probe: Were there any changes in your attitude? → If so, what were they?<br>○ Probe: Were there any changes in your interactions with your family or friends or co-workers? → If so, what were they?	

		• During or after voyaging, did you notice any changes in your emotional health?<br>○ Probes: Were there any changes in your mood?<br>○ Was there any change in your tolerance of other people or situations?<br><br>• During or after voyaging, did you notice any changes in your spiritual health?<br>○ Probes: Were there any changes in your cultural awareness? Were there any changes in your religious beliefs? Did you notice any difference in your level of tolerance or interest in your own cultural or spiritual beliefs?<br><br>• How long did any of the impacts that you noticed (positive or negative) last after you returned from a voyage?	
2.	Interpersonal	• Imagine when you first met your crewmates. Can you describe any changes, if any, in your connection to crewmates after voyaging with them?<br><br>• Please describe whether or not voyaging affected your previous connections with your family members (nuclear and extended), and if so, how.<br><br>• Please describe whether or not voyaging affected your previous interpersonal connections with other people, for instance with people at work, and if so, how.<br><br>• If there were changes, explain why you think voyaging influenced your interpersonal connections and relationships?	
3.	General	• Has voyaging changed you as a person? If so, what were any positive and negative changes?	

		• How does voyaging compare with other activities you have been involved with in terms of changing your enduring sense of health and wellness?<br>○ Probe: If you are an ocean person or sailor, did you notice a difference in how traditional voyaging affected you (positively or negatively) in comparison with those other ocean and/or vessel experiences?<br>• How would you suggest extending any positive impacts you may have had from voyaging, and limiting any negative impacts?	
4.	Education	• If you were asked to create a health education program, would you include anything that you do while voyaging or learned from voyaging?<br>• If yes, what would you include?<br>• Would any particular group most benefit from your voyaging health education program? If so, why?<br>• What would be the primary goal of your voyaging health education program?	
5.	Missed?	• Is there anything that you believe is vital to our understanding of Voyaging and Health & Wellness that has not been mentioned? → If so, please share your thoughts/manaʻo.	

That concludes our focus group. Thank you so much for coming and sharing your thoughts and opinions with us. We have a short evaluation form that we would like you to fill out if you have some time. If you have additional information that you did not get to say in the focus group, please feel free to write it on this evaluation form.
