# Supplementary material for: Exploring perspectives and insights of experienced voyagers on human health and Polynesian oceanic voyaging: A qualitative study
Source: PLoS One. 2024 Apr 15;19(4):e0296820. doi: 10.1371/journal.pone.0296820 (PMC11018278; doi:10.1371/journal.pone.0296820)
Supplement: S2 File — (PDF) [file pone.0296820.s002.pdf]

The Voyaging and Health Project
Voyaging Background and Demographic Questionnaire

1.a. Name ____
First Last

1.b. Today's Date ____ / ____ / ____

Please complete the following questionnaire to help us better understand the relationship between voyaging and health.

Demographic Information

2. Date of birth? (MM/DD/YYYY)

				/			/				

3. What is your gender? ☐ Male ☐ Female ☐ Other (specify): ____________

4. What is your current marital status?

☐ Married ☐ Single ☐ Never married ☐ Divorced/separated ☐ Widowed ☐ Other (specify): ____________

5.a. Which racial/ethnic group(s) do you belong to (Check ALL that apply)?

☐ Caucasian
☐ Chinese
☐ Filipino
☐ Native Hawaiian
☐ Japanese
☐ Korean
☐ Portuguese
☐ Micronesian (please specify): __________
☐ Pacific Islander (please specify): ________
☐ Other (please specify): ____________

5.b. Of the racial/ethnic group(s) you belong to, which do you *most closely identify* with (select ONLY ONE)?

☐ Caucasian
☐ Chinese
☐ Filipino
☐ Native Hawaiian
☐ Japanese
☐ Korean
☐ Portuguese
☐ Micronesian (please specify): __________
☐ Pacific Islander (please specify): ________
☐ Other (please specify): ____________

6. What is the highest degree or level of school you have completed?

☐ No high school diploma

☐ High school diploma

☐ Some college (please specify): ____________________

☐ Associate degree

☐ Bachelor degree

☐ Other (please specify): ____________________

Physical Activity Information

7. How would you rate yourself as to the amount of physical activity you get at work, compared with others of your age and gender?

☐	☐	☐	☐	☐	☐
Much more active	Somewhat more active	About the same	Somewhat less active	Much less active	Not Applicable

8. How would you rate yourself as to the amount of physical activity you get outside of work, compared with others of your age and gender?

☐	☐	☐	☐	☐	☐
Much more active	Somewhat more active	About the same	Somewhat less active	Much less active	Not Applicable

9. Do you regularly engage in strenuous exercise or hard physical labor?

☐ Yes
☐ No, skip question 11

10. Do you exercise or engage in physical labor at least three times a week?

☐ Yes
☐ No

Voyaging Information

11.a. In what year did you first get involved with traditional voyaging canoes? (YYYY)

11.b. In what year was your first "voyage" (lasting more than 24 hours and beginning or ending outside Hawaiʻi state waters) on a traditional canoe? (YYYY)

11.c. Where did your FIRST voyaging experience begin and end? (lasting more than 24 hours and beginning or ending outside Hawaiʻi state waters)

Location: From ________________________________ to ________________________________

11.d. How many separate "legs" of the WWV did you sail as voyaging crew

legs

11.e. To date, how many "voyages" have you sailed on a traditional voyaging canoe (waʻa kaulua)?

voyages

* “Voyaging” is defined as the act of ocean sailing and “living” on Polynesian traditional canoes (waʻa kaulua), which are designed for open-ocean sailing and which require the cooperation of other crew members to perform the act.
